# Flow-tub model: A modified bathtub flood model with hydraulic connectivity and path-based attenuation

**DOI:** 10.1016/j.mex.2023.102524

**Published:** 2023-12-15

**Authors:** Indraneel Kasmalkar, Dennis Wagenaar, Alina Bill-Weilandt, Jeanette Choong, Sonali Manimaran, Tian Ning Lim, Maricar Rabonza, David Lallemant

**Affiliations:** a Earth Observatory of Singapore, Nanyang Technological University, 639798, Singapore; b Asian School of the Environment, Nanyang Technological University, 639798, Singapore

**Keywords:** Flow-Tub, Coastal flooding, Bathtub model, Connectivity, Attenuation, Climate change

## Abstract

Global climate change and sea level rise are increasing the risks of flooding for coastal communities. Probabilistic coastal flood risk analysis at regional or global scales requires flood models with relatively low data requirements and low computational costs. Bathtub inundation models, which compute flood depth as the difference between water level and ground elevation, are well-suited for large-scale flood risk analysis. However, these models may overestimate floods because they do not capture some of the relevant underlying hydrodynamic processes that govern flood propagation on land.

We present Flow-Tub, a modified bathtub inundation model that integrates two hydrodynamic processes to improve the accuracy of the bathtub inundation model while retaining computational efficiency: hydraulic connectivity and path-based attenuation.

1. Hydraulic connectivity ensures that inundation is restricted to areas connected to the water source.
2. Path-based attenuation ensures that the modeled flood water depths are reduced along the flow paths to represent the effects of surface friction and the temporary nature of storm surges.

We validate the Flow-tub model against a hydrodynamic model. We also compare results of the bathtub model and the Flow-Tub model, highlighting the improved accuracy in the estimation of flood depths in the latter.

## Graphical abstract

**Figure:**
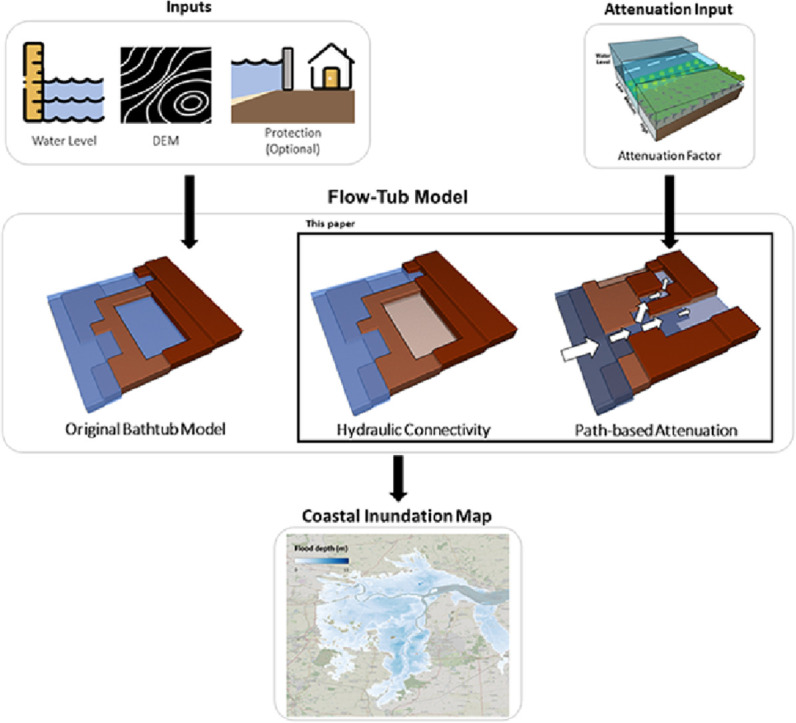

Specifications Table

**Table:** 

Subject area:	Environmental Science
More specific subject area:	Hydrology
Name of your method:	Flow-Tub
Name and reference of original method:	NOAA, 2012. Mapping Coastal Inundation Primer. Report. National Oceanic and Atmospheric Administration (NOAA) Coastal Services Centre. URL: https://coast.noaa.gov/data/digitalcoast/pdf/coastal-inundation-guidebook.pdf
Resource availability:	Python code for model available on Zenodo. 10.5281/zenodo.10012208

## Method details

### Background

Floods are the most frequently occurring natural hazards in the world, accounting for an average of 45 % of annual global disaster occurrences since the year 2000 [5]. In 2021, floods affected approximately 82 million people and resulted in approximately US$74 billion in economic losses [5]. Furthermore, climate change–driven sea-level rise is further increasing the risks posed by floods to coastal populations [6,^36^,42]. Around 40 % of the world's population live in coastal areas [22], and population growth along coasts is projected to outpace inland growth by 1.7 to 2 times [25], putting an estimated 1 billion people at risk by 2041–2060 [12].

Flood maps play a key role in understanding and managing coastal flood risks. They serve as crucial inputs in coastal flood risk assessments and have been used in several global exposure and vulnerability studies [e.g. 14,20,25,30,41]. Flood maps for local-level flood risk assessments are often generated by hydrodynamic models [10,^32^,35]. Hydrodynamic models are process-based models that perform water flow simulations numerically using either the full 3-D Navier-Stokes equations [26,21] or the simplified 2-D shallow water equations [3,^2^,^21^,32]. While hydrodynamic models offer high accuracy, they require high resolution local data and are computationally expensive, making them impractical for flood risk analysis on larger geographic scales [32,38], or probabilistic flood modeling requiring thousands of simulations.

In contrast to hydrodynamic models, bathtub models provide a computationally inexpensive alternative for modeling coastal floods at global and regional scales [15,^23^,^28^,33]. Often known as ‘static’, ‘planar’ or ‘equilibrium’ flood models, bathtub models compute flood depths by taking the difference between the water level and ground elevation [27]. Therefore, bathtub models sacrifice complex processes of water flow in favor of computational scalability.

**Fig. 1:**
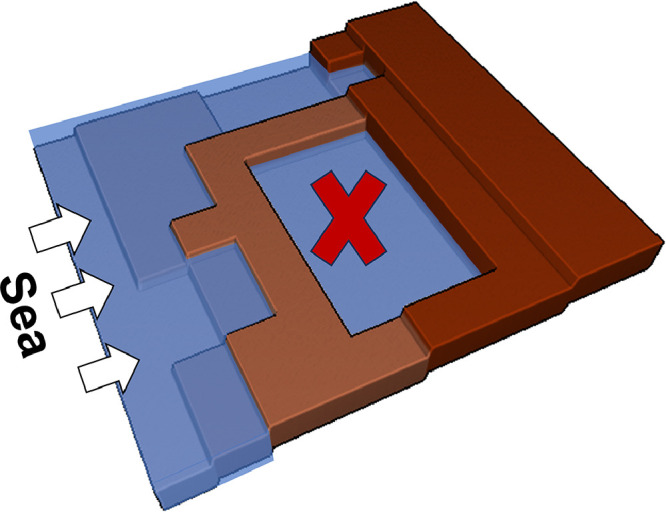
Standard bathtub model. The cross indicates an area that the model represents as flooded because of its low elevation even though the area has no viable path of water flow from the coastline.

However, standard bathtub models tend to overestimate flood depth and extent because they do not account for many of the relevant underlying hydrodynamic processes [27,^43^,44] (see Fig. 1). We identify two key processes, the absence of which may lead to a significant overestimation of flood risk. First, standard bathtub models fail to account for hydraulic connectivity [2,^11^,45]. As a result, they may flag low-lying areas as flooded, even though no viable pathway exists for water to reach these areas from the sea. This lack of consideration for connectivity can exaggerate flood risk projections, leading to the potential misallocation of disaster risk reduction resources [32]. Second, standard bathtub models do not account for friction along the flow path, as well as flood duration, and thus tend to overestimate coastal storm- driven flooding, especially in topographically flat regions [13,^24^,29]. For example, Vafeidis et al. [38] demonstrated that, in some cases, water level attenuation can reduce projected flood exposure and damages by 50 % or more.

To provide more accurate coastal flood mapping, whilst retaining some of the computational scalability of the original bathtub model, this paper presents a new model, Flow-Tub, which builds upon the original bathtub model by incorporating hydraulic connectivity and path-based attenuation. The model improves upon prior studies that include these processes by producing pathways of attenuated water flow that are consistent with the elevation, roughness, and protection level at each point along the pathway.

Despite the improvements, the Flow-Tub model is subject to general limitations from simplifying the physics of water flow across a complex surface. For example, complex geographies such as bay inlets and estuaries introduce additional hydrodynamic processes not captured by bathtub-based approaches. Further increasing the accuracy of flood risk mapping would require a hydrodynamic model along with more refined data inputs and greater computational resources compared to bathtub models.

### Hydraulic connectivity and path-based attenuation

The Flow-Tub model uses an efficient network-based algorithm to combine connectivity and attenuation at each step of water flow, thus providing flood maps with higher accuracy. We present the details of both features below. In this section, a pixel refers to the smallest unit of land represented in relevant model datasets. Recent global flood models have adopted pixel resolutions of 30 m [4,16] or 90 m [20].

**Fig. 2:**
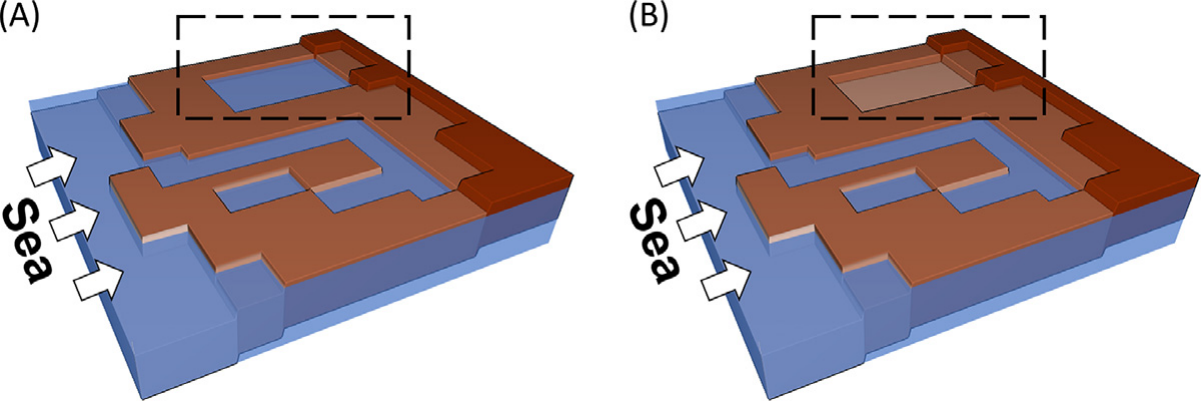
Connectivity in bathtub models. (A) Standard bathtub model. (B) Bathtub model with connectivity. The dashed rectangle shows a low-lying region that standard bathtub models may misrepresent as flooded. Connectivity allows for models to test for viable flow pathways for water into such regions.

Hydraulic connectivity, as shown in Fig. 2, imposes the condition that, for a pixel to be deemed as flooded, there must be a path of flooded pixels leading to the pixel. Hydraulic connectivity has been previously highlighted as an important feature for bathtub models and has been implemented in most prior models (see Table 1). Since the connectivity of a pixel relies on the connectivity of its neighboring pixels, the feature of connectivity introduces serial computations in the otherwise parallel nature of standard bathtub models where we simply compare water levels to the elevation values at each pixel.

**Table 1:** Overview of bathtub models, with and without attenuation and hydraulic connectivity.

Reference	Hydraulic connectivity	Attenuation
Hanson et al. [14]	No.	No.
NOAA [27]	No, but recommend manual edits to account for connectivity.	No.
Hooijer and Vernimmen [17]	No.	No.
Vernimmen and Hooijer [41]	No.	No.
Jongman et al. [18]	Yes.	No.
Muis et al. [24], Kirezci et al. [19]	Yes.	No.
Kulp and Strauss [20]	Yes.	No.
Dasgupta et al. [7]	No.	Yes, attenuation along a straight line from coast land inwards.
Tiggeloven et al. [37]	Yes.	Yes, attenuation along a straight line from coast land inwards.
Vafeidis et al. [38]	Yes.	Yes, attenuation along a straight line from coast land inwards.
Van Coppenolle and Temmerman [40]	Yes.	Yes, attenuation along the path of least attenuation.
**Current paper**	**Yes.**	**Yes, attenuation along the path of water flow.**

**Fig. 3:**
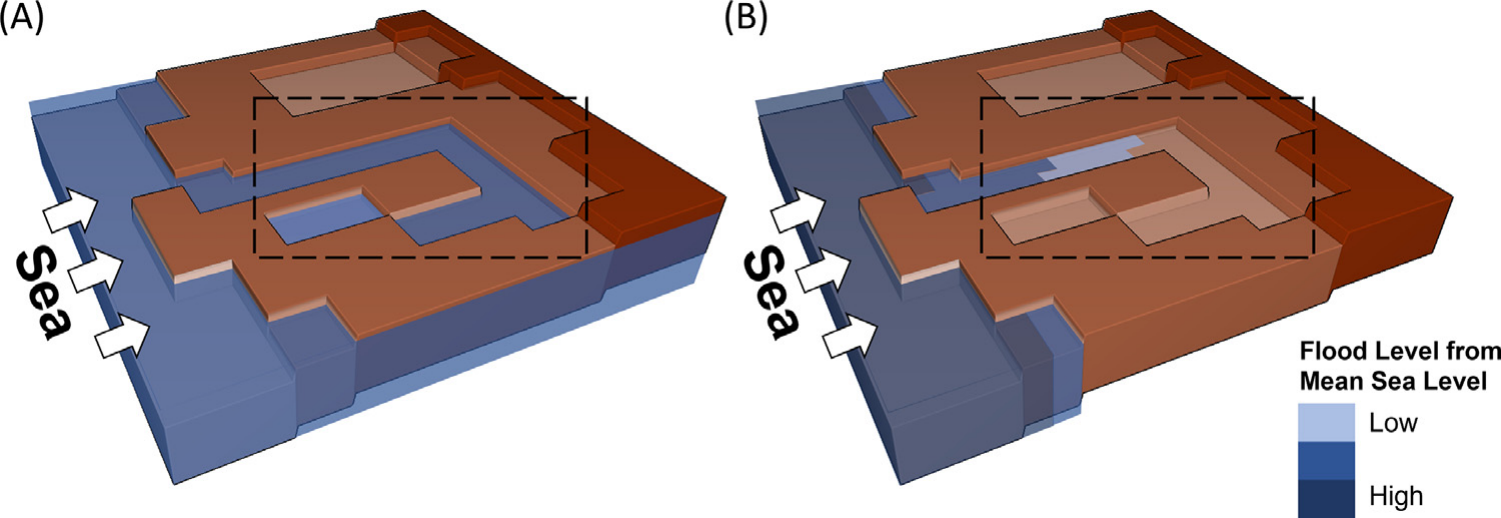
Attenuation in bathtub models. (A) Bathtub model without attenuation. (B) Bathtub model with attenuation. The dashed rectangle shows a region where attenuation reduces the depth and extent of the flood.

Attenuation refers to the reduction of water depth as water travels inland (see Fig. 3). Attenuation is primarily a consequence of limited time duration of high water levels caused by events such as astronomical tides or storm surges. During the limited time of the event, factors such as friction limit the overall flood extent caused by the event [38,^37^,40].

Attenuation is modeled by an attenuation factor, namely the reduction of water level (in cm) per unit of distance traveled (per km), which can be specified per pixel or as a uniform value across all pixels. Once an attenuation factor is set (per pixel or uniformly), the attenuation depends on the path over which attenuation occurs. Some coastal flood models use the straight-line shortest path from the coast to any given point and compute the total attenuation for the point as the integration of the attenuation factor along the path [37]. While this approach allows for simplified computation, it may substantially underestimate the attenuation given that water may flow along much longer, non-linear paths. Other models improve upon the straight-line approach by calculating paths of least attenuation. In this case, the models use a cost-distance algorithm to optimize for paths of least attenuation, and they assume that the water would flow along the path [9,^39^,44]. While the path of least attenuation presents a substantial improvement over straight-line paths, the actual path of water flow may be notably different from the path of least attenuation. Local elevation and protection profiles may cause water to travel along alternate flow paths, which would cause the path-of-least-attenuation to underestimate the actual attenuation for a given pixel. Computing flow paths while consistently accounting for attenuation requires a path-based attenuation approach, where, at each step of flow propagation, the attenuated water level is compared against ground elevation and estimated protection. The Flow-Tub model utilizes such a path-based attenuation approach, described further in the Algorithm section.

## Model inputs

For a given region of analysis, the model requires four input datasets: elevation, water level, attenuation factor(s), and flood protection. The elevation dataset, also known as a Digital Elevation Model (DEM) allows the model to identify low-lying areas that may potentially be flooded. For the analyses within the current paper, we use the Forest And Buildings removed Copernicus DEM (FABDEM), developed by Hawker et al. [16], available at 30 m resolution globally in GeoTiff format.

Water level refers to the height of the water surface relative to a local coordinate system. The water level dataset indicates the height of the water surface at points along the coast, corresponding to a given extreme coastal storm event or for a specified flood return period. The study by Dullaart et al. [8] provides global storm surge water levels for coastal points for a range of return periods. For the current paper, we prepare global-scale water level datasets by spatially interpolating the coastal storm surge return-period point-data of Dullaart et al. [8] into GeoTiff format. The interpolation procedure is done via the ArcGIS ‘Interpolate Points’ tool.

Interpolation of coastal water levels into inland regions overestimates the inland water levels because there is an implicit assumption that the inland water levels have sufficient amount of time to equalize with the coastal water levels. This assumption is not suitable for episodic coastal storm surges, especially in places with high surface friction. Hence the need for attenuation. Our model can take a GeoTiff raster file with an attenuation factor per pixel. While such attenuation rasters may be available for local regions, to our knowledge, no such attenuation raster has been made publicly available for large regions. For the purposes of regional-scale coastal flood risk model, we use a uniform attenuation factor value of 20 cm/km. The order of magnitude of the factor is consistent with prior models [8]. We present further details in the Method validation: Sensitivity and error section.

Flood protection data is critical for flood risk analysis, the absence of which can cause substantial overestimation of flooding [31]. For large-scale and global analyses, obtaining relevant flood protection data for each region presents a critical challenge. We prepare a global coastal flood protection dataset using the method and procedure outlined in Scussolini et al. [31]. We estimate the relevant return period up to which a region is protected against, based on the frequency of flooding and the wealth of the region. The protected return period is converted into water levels of protection using the global return period water levels provided in Dullaart et al. [8]. Hence, our flood protection dataset provides approximate water level thresholds for each area that must be exceeded for the areas to be flooded. The protection dataset is stored in GeoTiff format.

## Algorithm

The Flow-Tub model computes water depth as follows:

(1) $$w_{d}=\left\{ \begin{array}{cc} \max\left(w_{l}-z-a,\;0\right) & w_{l}\geq P\\ 0 & w_{l}<P \end{array}\right.$$

where *w_d_* is the water depth at pixel X, *w_l_* is the water level at pixel X interpolated from coastal water levels, *z* is the elevation at pixel X, *a* is the attenuation of the water level resulting from the flow pathway of the water from the coast to pixel X, and P is the flood protection threshold. In other words, the water depth at a pixel is equal to the difference between the water level and the elevation, while accounting for attenuation, provided that the water level at the point exceeds the local protection measure.

**Fig. 4:**
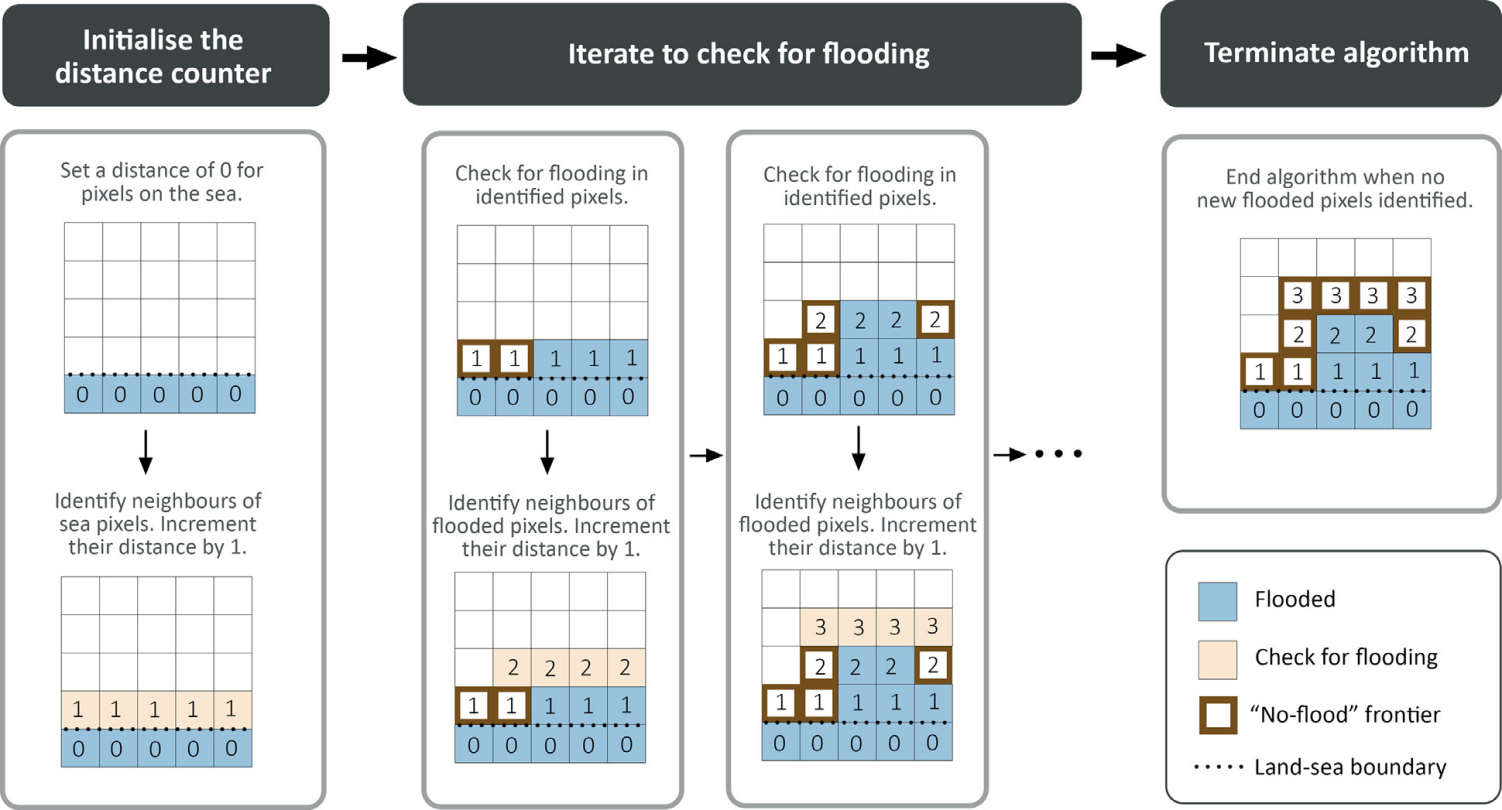
Schematic diagram of the Flow-Tub model algorithm.

Our bathtub model specifically improves over the computation of *w_l_* and *a* by accounting for hydraulic connectivity and path-based attenuation. We present the algorithm below, along with a schematic diagram in Fig. 4.

1. START: For all pixels on the coastline, assign distance zero and total attenuation zero. Define a distance counter that is initially set to 0. This represents the current pixels under consideration.
2. Among the pixels with distance equal to distance counter, if the attenuated water level *w_l_*−*a* exceeds protection *P* and the ground elevation *z*, set the flood depth as per Eq. (1). The attenuation *a* equals 0 for the coastline pixels.
3. For each considered pixel that is flooded (*w_d_ >* 0), take all its neighboring pixels that have not been assigned a distance yet, including diagonal neighbors. Assign them distance 1 plus the current distance counter. Set attenuation of these neighboring pixel equal to the attenuation factor for the current pixel multiplied by the distance, i.e. the resolution of the pixel. If two pixels have a common neighboring pixel, the lower attenuation value is recorded.
4. Increase the distance counter by 1 and repeat from Step 2 until there are no new pixels left.

Existing Geographical Information System (GIS) tools such as ArcGIS or QGIS do not have the requisite subroutines to implement the above algorithm. Therefore, we have implemented the algorithm in Python (please see Resource Availability). The python code also provides an optional functionality where diagonally neighboring pixels are considered to be one-and-half-unit distance apart in the calculation of attenuation, as opposed to the unit distance for side neighbors. For the above algorithm, the python code takes GeoTiff raster files as inputs and produces flood maps in GeoTiff raster format, which can be viewed using GIS software.

### Method validation: sensitivity and error

To estimate the error of the Flow-Tub model, we compare results against those of a hydrodynamic model. We choose the San Francisco Bay Area, California, USA as the region of comparison because of its relatively large area, its varied geography complex topography around the bay, and the availability of a high-resolution hydrodynamic flood model [1]. The flood maps for the region are generated by the United Stated Geological Survey Coastal Modeling System [1] as part of the Our Coast Our Future (OCOF) project. We compare the 1-in-100-year return period coastal flood maps of OCOF against those generated by the Flow-Tub model.

For a proper comparison of the two flood maps, we ensure that both maps are generated from the same underlying DEM. Inaccuracies in the DEM may introduce substantial errors in the outputs of flood models [1]. The OCOF maps were generated using the United States Geological Survey 10-m LiDAR-based DEM [34].

Discrepancies in the coastal storm surge water levels between the two models may also introduce confounding errors. Therefore, we ensure that both models have the same water levels at all coastline pixels. For this purpose, we use a modified version of the Flow-Tub algorithm, where the model only takes water level input at each coastline pixel and successively propagates the water level values to neighboring pixels while accounting for attenuation. We then run the Flow-Tub model with the coastline pixel water level values extracted from the OCOF maps.

The choice of attenuation factor may introduce notable error in the Flow-Tub model results as well. The attenuation factor varies with geography and land use and is difficult to estimate [8]. For the purposes of large-scale coastal flood mapping, bathtub models use an approximated uniform attenuation factor across all land pixels [37,38]. The Flow-Tub model uses a uniform attenuation factor of 20 cm/km. To understand how model error may vary with changes in the attenuation factor, we perform a sensitivity analysis against the attenuation factor with values of 5, 10, 20, 30, 40, 50, 80, and 100 cm/km.

We show the results of our error and sensitivity analysis in Table 2. For each pixel, we compute the OCOF flood depth against the flood depth outputted by the Flow-Tub model. We summarize the flood depth errors using the Mean Absolute Error (MAE) and the Root Mean Square Error (RMSE). We compute MAE and RMSE for all pixels, and separately for all flooded pixels. By all pixels, we refer to all pixels in the administrative region of the San Francisco Bay Area. By flooded pixels, we refer to the pixels deemed as flooded in the OCOF flood maps.

**Table 2:** Comparison of the Our Coast Our Future (OCOF) 1-in-100-year return period flood depth map with our model flood depth maps across a range of attenuation factors. Our model is run with the same coastline water levels as the OCOF map. Mean Absolute Error (MAE) and Root Mean Square Error (RMSE) for all pixels (flooded or otherwise) and flooded pixels (as per the benchmark OCOF map). The bolded line highlights the default model attenuation factor of 20 cm/km.

Attenuation (cm/km)	MAE (all pixels) (m)	RMSE (all pixels) (m)	MAE (flooded pixels) (m)	RMSE (flooded pixels) (m)
5	0.024	0.176	0.343	0.582
10	0.020	0.159	0.331	0.556
**20**	**0.017**	**0.138**	**0.319**	**0.525**
30	0.015	0.125	0.334	0.530
40	0.014	0.119	0.351	0.544
50	0.013	0.114	0.381	0.573
80	0.013	0.110	0.442	0.634
100	0.013	0.109	0.478	0.666

As shown in Table 2, the MAE for all pixels (flooded or otherwise) is within the range 0.01 - 0.03 m, suggesting good agreement. On flooded pixels specifically, the MAE is within 0.3 to 0.5 m, also suggesting that the Flow-Tub models have relatively low error. The MAE of all pixels decreases with increasing attenuation, suggesting that higher attenuation reduces incorrectly flooded pixels. However, the MAE and for the flooded pixels (as per the benchmark OCOF map) are lowest for the attenuation factor 20 cm/km, with an error 0.32 m. The RMSE values show similar patterns.

**Fig. 5:**
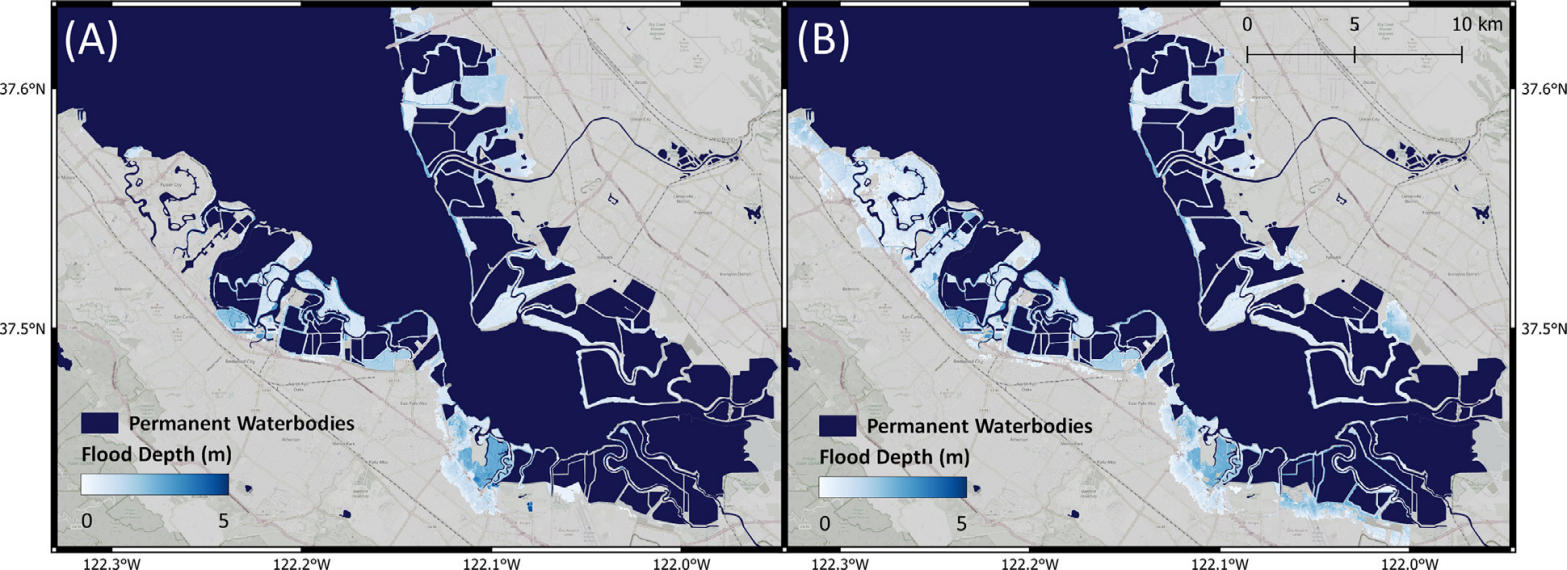
Coastal flood maps for the 1-in-100-yr return period, (A) The Our Coast Our Future flood map. (B) Flood map generated by the Flow-Tub model, with attenuation factor of 20 cm/km.

In Fig. 5, we show the OCOF flood map and the model output flood map for the attenuation factor of 20 cm/km. A visual inspection suggests that the flood maps are highly similar. The area in the north-east of the map is deemed as flooded in the Flow-Tub flood map, but not in the OCOF flood map. The discrepancy arises from the fact that the OCOF flood maps incorporate the latest plans related to flood protection in Foster City, California [1], while the Flow-Tub flood maps only incorporate estimated protection levels derived from Scussolini et al. [31]. For easier comparison of the two flood maps, we provide a map of the difference in flood depths in the Supplementary Materials. The overall results suggest that the Flow-Tub model and hydrodynamic model produce notably similar flood depths.

### Method validation: improvement over standard bathtub models

**Fig. 6:**
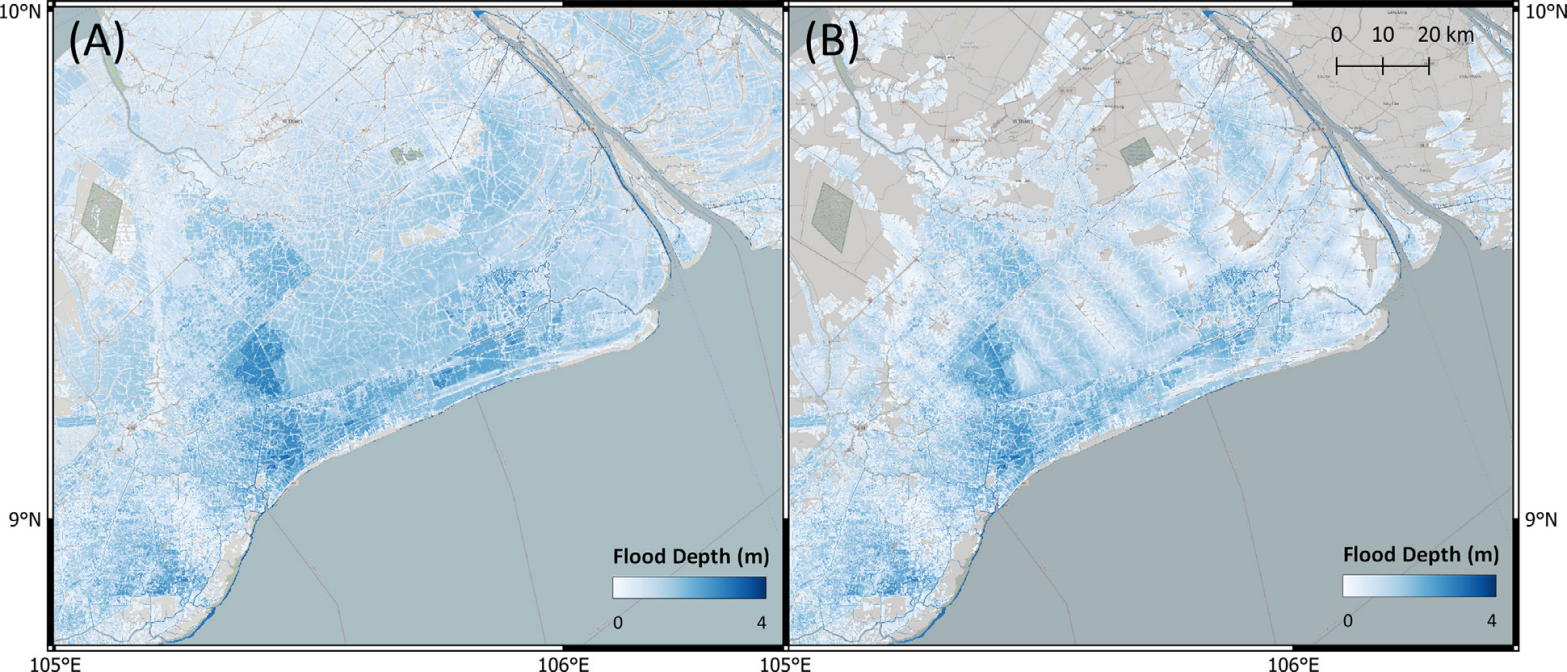
Floods maps for Bac Lieu, Vietnam, generated by (A) the standard bathtub model and (B) the bathtub model with connectivity and path-based attenuation. The flooded area is shaded in blue and scaled by the flood depth. These models were run for the year 2020 at a 1-in-1000 year return period.

**Fig. 7:**
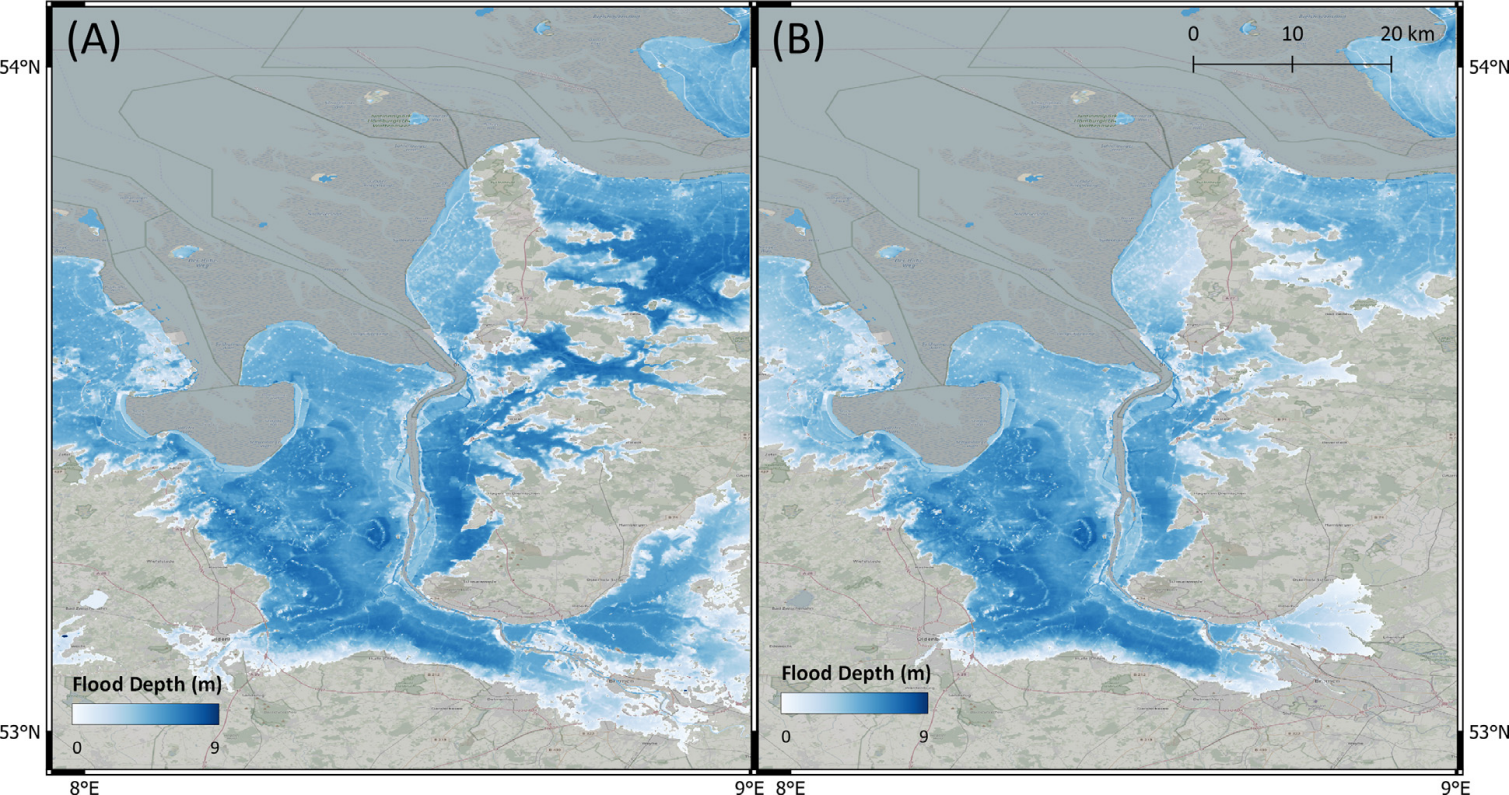
Floods maps for Bremerhaven, Germany, generated by (A) the standard bathtub model and (B) the bathtub model with connectivity and attenuation. The flooded area is shaded in blue and scaled by the flood depth. These models were run for the year 2020 at a 1-in-1000-year return period.

**Fig. 8:**
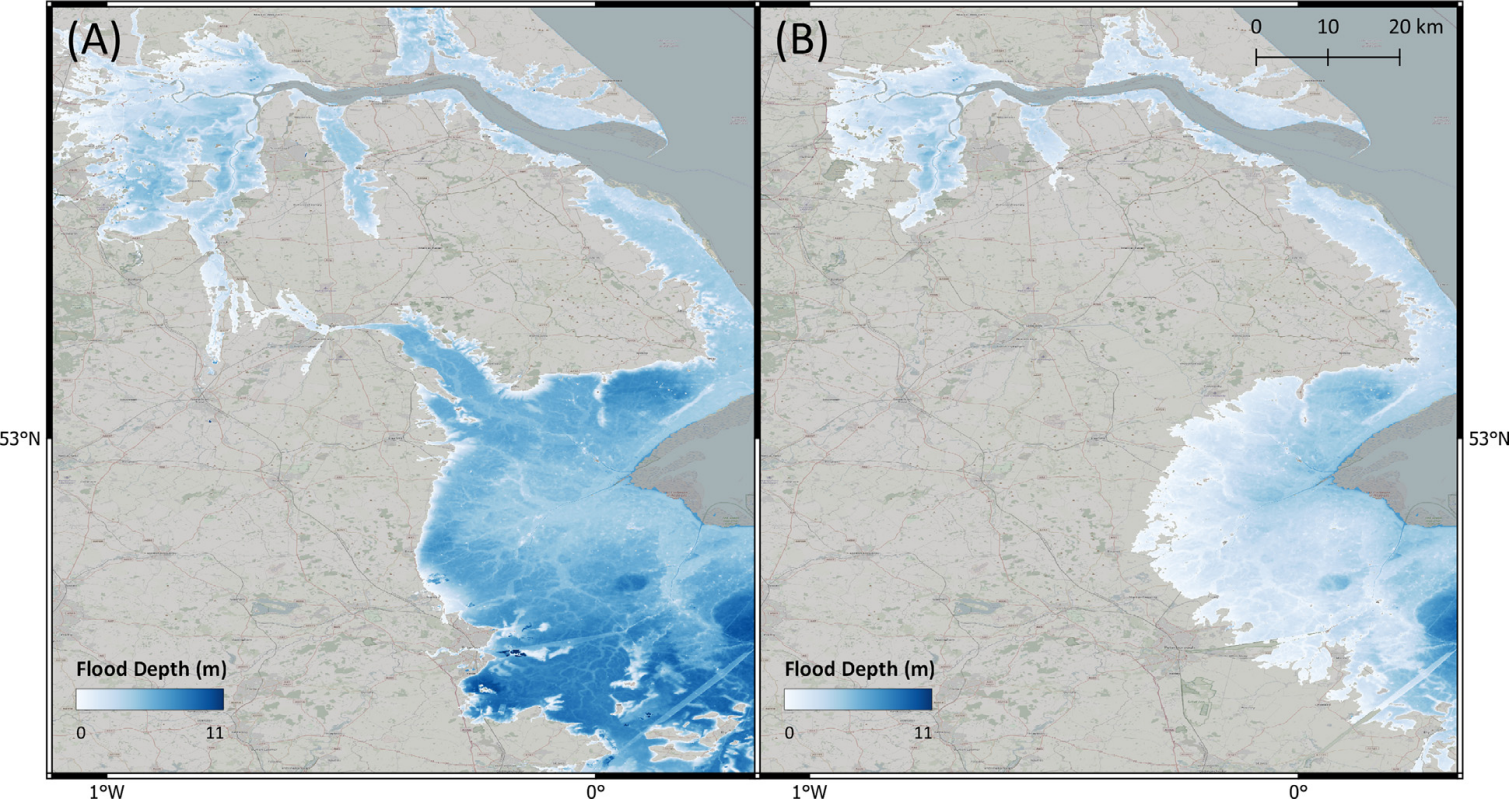
Floods maps for Hull, United Kingdom, generated by (A) the standard bathtub model and (B) the bathtub model with connectivity and attenuation. The flooded area is shaded in blue and scaled by the flood depth. These models were run for the year 2020 at a 1-in-1000-year return period.

**Fig. 9:**
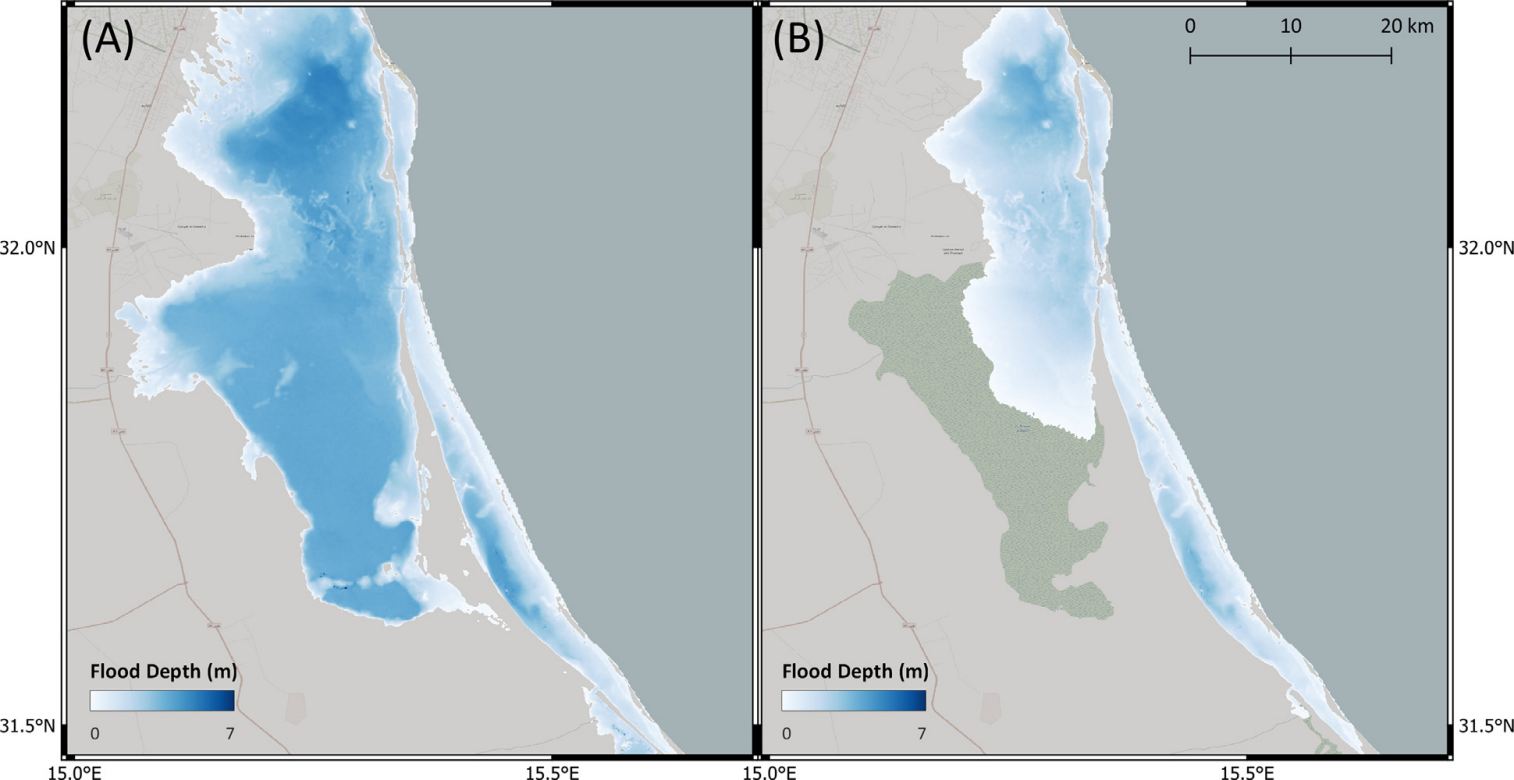
Floods maps for Misrata, Libya, generated by (A) the standard bathtub model and (B) the bathtub model with connectivity and attenuation. The flooded area is shaded in blue and scaled by the flood depth. These models were run for the year 2020 at a 1-in-1000-year return period.

In Table 3, we present results of the standard bathtub model and the Flow-Tub model for four regions. Both models are initialized with the parameters and inputs as laid out in the Model Inputs section. The results and corresponding 1-in-1000-year return period flood maps (Table 3 and Fig. 6, Fig. 7, Fig. 8, Fig. 9) demonstrate that connectivity and path-based attenuation reduce flood extents and volumes compared to standard bathtub models as well as models with just connectivity. With connectivity, inland areas that are below future projected water levels but disconnected from the coastline are not deemed as flooded. This is especially evident in the bottom-left corner for the flood maps for Bremerhaven, Germany (Fig. 7). Similarly, accounting for path-based attenuation reduces projected flooding inland. This is seen clearly in the case of Hull, United Kingdom (Fig. 8), in which the flood extent is much smaller inland when attenuation is factored in.

**Table 3:** Comparison of the predicted flood area and volume at various locations produced by the standard bathtub model, bathtub model with connectivity, and bathtub model with connectivity and path-based attenuation. These models were run for the year 2020 at a 1-in-1000-year return period. The total map area considered refers to the total area over which the flood models were run, depending on the bounding coordinates specified.

Location	Total map area considered (km^2^)	Bathtub		Bathtub + Connectivity		Bathtub + Connectivity + Attenuation	
		Flood area (km^2^)	Flood volume (km^3^)	Flood area (km^2^)	Flood volume (km^3^)	Flood area (km^2^)	Flood volume (km^3^)
Bac Lieu, Vietnam	111,529	26,159	22.25	24,745	21.95	15,140	13.60
Bremerhaven, Germany	148,706	21,983	82.55	21,281	80.63	15,999	57.97
Hull, United Kingdom	148,706	11,366	46.69	11,152	49.49	7,478	20.34
Misrata, Libya	74,352	2232	5.93	2050	5.43	950	1.41

The validation results suggest that the Flow-Tub model, with its features of hydraulic connectivity and path-based attenuation, provides notably greater accuracy over standard bathtub inundation models, and is therefore a suitable candidate for large-scale coastal flood risk mapping.

## Ethics statements

We comply with the relevant ethical guidelines of MethodsX.

## CRediT authorship contribution statement

**Indraneel Kasmalkar:** Methodology, Software, Validation, Formal analysis, Data curation, Supervision, Writing – original draft, Writing – review & editing. **Dennis Wagenaar:** Conceptualization, Methodology, Investigation, Data curation, Writing – review & editing. **Alina Bill-Weilandt:** Investigation, Writing – original draft, Writing – review & editing. **Jeanette Choong:** Investigation, Writing – original draft, Writing – review & editing. **Sonali Manimaran:** Formal analysis, Visualization, Writing – original draft, Writing – review & editing. **Tian Ning Lim:** Visualization, Writing – review & editing. **Maricar Rabonza:** Visualization, Writing – original draft, Writing – review & editing. **David Lallemant:** Supervision, Project administration, Funding acquisition, Writing – review & editing.

## Declaration of Competing Interest

The authors declare that they have no known competing financial interests or personal relationships that could have appeared to influence the work reported in this paper.

## Data Availability

The Python code for the model is available at 10.5281/zenodo.10012208.

## Appendix Supplementary materials

Image, application 1
