# Supplementary material for: Flow-tub model: A modified bathtub flood model with hydraulic connectivity and path-based attenuation
Source: MethodsX. 2023 Dec 15;12:102524. doi: 10.1016/j.mex.2023.102524 (PMC10772817; doi:10.1016/j.mex.2023.102524)
Supplement: Supplementary file 1 [file mmc1.docx]

**Supplementary Materials for “Flow-Tub model: a modified bathtub flood model with hydraulic connectivity and path-based attenuation.”**

| 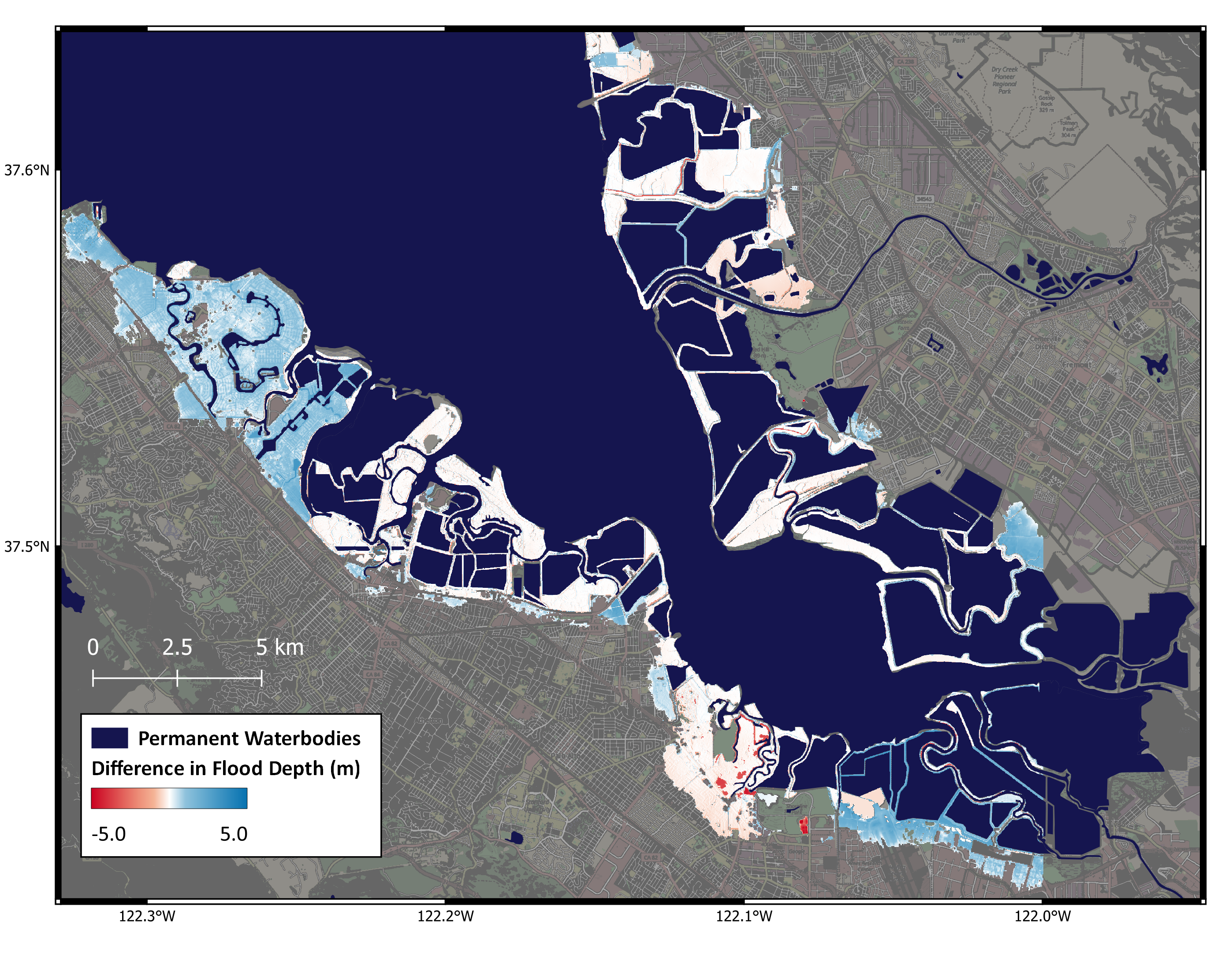 |
| --- |
| Supplementary Figure 1: Difference in flood depths of the 1-in-100-yr return period flood map generated by the Flow-Tub model with attenuation factor of 20cm/km, and the 1-in-100-yr return period Our Coast Our Future flood map. The figure represents a difference in the Figures 5(B) and 5(A) from the main article. |
